# PAL SPME Arrow—evaluation of a novel solid-phase microextraction device for freely dissolved PAHs in water

**DOI:** 10.1007/s00216-015-9187-z

**Published:** 2015-12-16

**Authors:** Andreas Kremser, Maik A. Jochmann, Torsten C. Schmidt

**Affiliations:** Instrumental Analytical Chemistry, University of Duisburg-Essen, Universitätsstraße 5, 45141 Essen, Germany

**Keywords:** PAL SPME Arrow, SPME, Microextraction, PDMS, PAH

## Abstract

After more than 25 years, solid-phase microextraction (SPME) has gained widespread acceptance as a well-automatable and flexible microextraction technique, while its instrumental basis remained mostly unchanged. The novel PAL (*P*rep *A*nd *L*oad solution) SPME Arrow combines the advantages of SPME with the benefits of extraction techniques providing larger sorption phase volumes such as stir bar sorptive extraction (SBSE). It thereby avoids the inherent drawbacks of both techniques such as limitations in method automation in the case of SBSE, as well as the small sorption phase volumes and the lacking fiber robustness of classical SPME fibers. This new design is based on a robust stainless steel backbone, carrying, the screw connection to the PAL sampler, the enlarged sorption phase, and an arrow-shaped tip for conservative penetration of septa (hence the name). An outer capillary encloses this phase apart from enrichment and desorption processes and rests against the tip during transfer and penetrations, resulting in a homogeneously closed device. Here, we present an evaluation and a comparison of the novel PAL SPME Arrow with classical SPME fibers, extracting polycyclic aromatic hydrocarbons (PAHs) as model analytes, from the freely dissolved fraction in lab water and groundwater via direct immersion using polydimethylsiloxane (PDMS) as common sorption phase material. Limits of detection, repeatabilities, and extraction yields were determined for the PAL SPME Arrow and compared to data of classical SPME fibers and SBSE bars. Results indicate a significant benefit in extraction efficiency due to the larger sorption phase volume. It is accompanied by faultless mechanical robustness and thus better reliability, especially in case of prolonged, unattended, and automated operation. As an exemplary application, the water-soluble fraction of PAHs and derivatives in a roofing felt sample was quantified.

**Electronic supplementary material:**

The online version of this article (doi:10.1007/s00216-015-9187-z) contains supplementary material, which is available to authorized users.

**Graphical Abstract:**
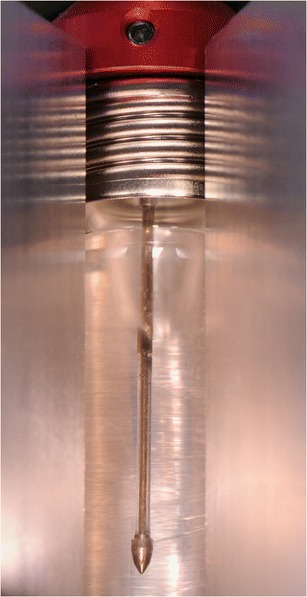
Picture of a PAL SPME Arrow during extraction of a stirred water sample

## Introduction

Solid-phase microextraction (SPME) was developed by Belardi and Pawliszyn in 1989 [1] and is nowadays the most popular and most frequently used microextraction technique [2]. The reasons for this popularity are its operational simplicity, short extraction times, possibility of a fully automated operation, avoidance of organic solvents [3], as well as its direct and straightforward thermodesorption into a gas chromatographic system. Furthermore, SPME combines matrix separation of analytes with a concentrating step [4] and can be used for in situ, in-field, and even in vivo sampling [2, 5, 6]. However, apart from many advantages, it also has drawbacks, including the limited mechanical robustness of the fiber [7–10] and the rather small sorption phase volume of the commercially available fibers [2, 8, 11].

In order to overcome especially the latter disadvantage, the SPME-related technique stir bar sorptive extraction (SBSE) was developed. SBSE provides a significantly larger extraction phase in the order of 100 μL compared to about 1 μL with classical SPME, but loses the advantage of full automation, as the SBSE bar has to be recovered from the sample, dried, and introduced into a special thermodesorption unit in a manual process.

**Fig. 1:**
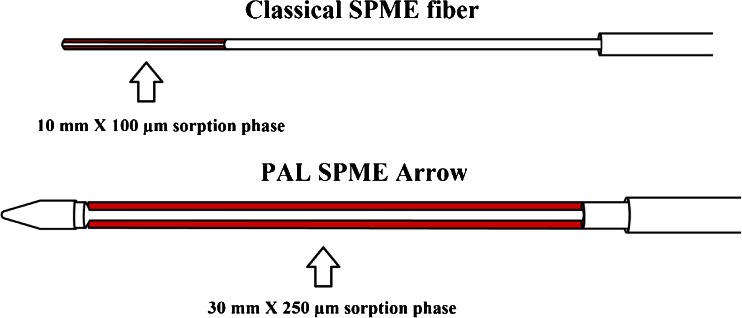
Sketch of a classical SPME fiber and a novel PAL SPME Arrow. The SPME fiber possesses a 100-μm × 10-mm, 0.6-μL sorption phase. The PAL SPME Arrow is equipped with a 250-μm × 30-mm, 15.3-μL sorption phase, respectively, has a stainless steel inner core with a diameter of 0.4 mm, and an overall external diameter of 1.5 mm

Recently, a novel SPME-related extraction device named PAL (*P*rep *A*nd *L*oad solution) SPME Arrow was developed. As the first alternative in this field to be based on a completely redesigned, automatable fiber, it aims at combining the advantages of the classical SPME fiber and the SBSE, while remediating the main inherent disadvantages of these techniques. It is presented in Fig. 1 alongside a classical SPME fiber, and its properties will be thoroughly discussed in the “Results and discussion” section.

Polycyclic aromatic hydrocarbons (PAHs) are abundant environmental contaminants originating from both anthropogenic as well as natural sources, which typically involve incomplete combustion processes such as forest fires or burning of fossil fuels [5, 12]. PAHs are also contained in bitumen-related products that are used in various fields of construction, especially due to their hydrophobic properties, which make them a widespread choice for waterproofing applications [13]. While the fumes and vapors that originate from production and handling of such materials are already suspected to represent occupational risks in terms of exposure to PAHs [13], the leaching of the latter compounds into runoff water was mostly neglected in the past, often due to insufficient detection limits of the analytical methods [14].

Typical SPME LODs and LOQs for measurements of PAH in water are in the nanogram-per-liter range, depending on utilized sorption phase and analytical conditions [15]. In this context of increasing analytical demands, PAHs were used as representative and well comparable analytes in order to determine to which extent PAL SPME Arrow surpasses limitations of classical SPME fibers without compromising original SPME advantages.

Polydimethylsiloxane (PDMS) was used as common sorption phase material [16] because, just like the aforementioned analytes, it enables effective comparison of results with literature.

## Experimental section

### Reagents and materials

Optimization and calibration were carried out by using a PAH standard (SV Calibration Mix #5/610 PAH) purchased from Restek (Bellefonte, PA). The standard contains 16 PAHs in methylene chloride at a concentration of 2 g L^−1^, respectively (see Table S1 in the electronic supplementary material (ESM)). Analytical-grade methanol (KMF Laborchemie, Lohmar, Germany) and lab water from a PURELAB Ultra analytic water purification system (ELGA LabWater, Celle, Germany) were used as solvents for stock, standard, and sample preparation. In the case of groundwater samples, the water was kindly supplied by LINEG (Kamp-Lintfort, Germany) and filtered through medium-dense MN 615 cellulose filters with a thickness of 0.16 mm and a surface weight of 70 g m^−2^, which were obtained from Macherey-Nagel (Düren, Germany). G200 DD sanded roofing felt according to EN 13969 and EN 14967 was purchased at a Hornbach building supply store (Essen, Germany).

### Standard solutions

From the PAH calibration mix, a methanolic stock solution with a concentration of 1 mg L^−1^ was prepared and stored in a 20-mL amber screw-cap headspace vial, with silicone/PTFE septa and no headspace (BGB Analytik, Boeckten, Switzerland), in the refrigerator at 4 °C. From this stock solution, aqueous standard dilutions were prepared and stored in the same manner. Hamilton glass syringes (Hamilton, Bonaduz, Switzerland) and Blaubrand^®^ bulb pipettes (Brand, Wertheim, Germany) were used for stock, dilution standard, and sample preparation.

The PDMS tubes which were used as extraction phases for PAL SPME Arrows were also obtained from BGB Analytik.

Roofing felt samples were prepared by cutting the material into pieces of 2 mm × 4 cm (approx. 300 mg) and adding one of these pieces to vials containing 19 mL of lab water. Pieces were deliberately used as a whole since further disintegration would have resulted in a larger total surface area of the material and therefore an overestimation of PAH leaching into water.

Since PAHs readily adsorb to almost any available surface and are thereby lost to solid-phase extraction processes, it is reasonable to calculate their equilibrium ratios that are adsorbed to the surfaces available in the prepared samples in order to avoid biased results [17].

Partitioning of analytes into the headspace was calculated [18] as, e.g., 0.16 % for naphthalene, which is the most volatile PAH. Analyte loss due to sorption to glassware was calculated as well [19], with adsorbed analyte fractions of, e.g., 0.3 % in case of pyrene. Sorption of analytes to the PTFE septa of sample vial caps was the strongest influence in this context, with an equilibrium value of 3.8 % for pyrene [17].

Therefore, 3.8 % can be considered to be the maximum value here, resulting in a total loss of adsorbed analytes below 5 %, which was neglected during the further course of this study. In order to ensure proper sample equilibration prior to measurement series, samples were incubated at room temperature for at least 24 h prior to extraction.

Samples were stirred with self-constructed stir bars prepared from 1.5 × 10-mm iron cylinder bolts enclosed in fused silica.

### GC/MS instrumentation and parameters

All analyses were carried out on a Shimadzu GCMS-QP2010 Ultra (Shimadzu Deutschland GmbH, Duisburg, Germany). Thermal desorption of the extracted analytes was carried out using a split/splitless injector, which was set to a temperature of 280 °C. The injector was equipped with a Restek (2 mm i.d. × 5 mm o.d. × 95 mm length) splitless liner (BGB Analytik, Boeckten, Switzerland). The thermal desorption time was 5 min, and after a splitless time of 6 min, the split ratio was set to 10:1.

Analyte separation was accomplished on a 30-m × 0.25-mm Rxi^®^-PAH column (Restek, Bellefonte, PA) with a 0.1-μm film thickness. As carrier gas, helium 5.0 (Air Liquide, Oberhausen, Germany) with a flow of 1.5 mL min^−1^ was used. The GC temperature program started with a 5-min standby at 40 °C, followed by a first temperature ramp of 50 °C min^−1^ up to 110 °C, a second ramp of 5 °C min^−1^ to 240 °C, and a third ramp of 50 °C min^−1^ to a final temperature of 320 °C, which was maintained for 5 min for cleanup purposes. The transfer line and ion source were both set to 250 °C, respectively. Retention times varied between 8.70 and 49.48 min for naphthalene-d_8_ and benzo(ghi)perylene (see Table S2 in ESM), respectively.

In accordance with literature [20], the chosen chromatographic conditions enabled sufficient separation of all target compounds. A resulting chromatogram is included in the ESM (Fig. S4), as well as detailed mass spectrometric detection parameters.

### Extraction procedure

Samples were extracted by a PAL RTC autosampler, which was equipped with SPME fibers (100 μm × 10 mm, 0.6 μL) and PAL SPME Arrows (250 μm × 20 mm, 10.2 μL) (all from CTC Analytics AG, Zwingen, Switzerland). The 20-mm-long sorption phase was chosen for PAL SPME Arrow to facilitate constant and complete submersion during extraction.

Due to the larger diameter of PAL SPME Arrow in contrast to traditional SPME fibers, the openings of the PAL tool needle guide, the GC injector, and the SPME fiber conditioning station had to be widened to approximately 1.8 mm.

Samples were stored in their tray at room temperature (23 °C). Prior to extraction, they were transferred to a self-constructed stirring station based on an IKA-Mag RCT basic (IKA-Werke GmbH & CO KG, Staufen, Germany). In this station, samples were continuously stirred at 1500 rounds per minute (rpm) and 35 °C, first for a temperature pre-equilibration time of 10 min and afterwards during sample extraction. Simultaneous to the first 5 min of sample pre-equilibration time, the SPME fiber or PAL SPME Arrow was preconditioned in the SPME fiber conditioning station at 200 °C under a stream of nitrogen 5.0.

After the sample pre-equilibration time, the sample vials’ septa were pierced by the fiber and the sorption phase was immersed into the continuously stirred sample for 70 min. The sample vial penetration depth was thereby set to 55 mm, in order to ensure constant and complete immersion of the sorption phase.

Once extraction was completed, the fiber was transferred into the GC injector for thermal desorption at 280 °C. Subsequently, it was cleaned for 15 min in the SPME fiber conditioning station at 200 °C. The PAL RTC sequence was interlocked so that the subsequent equilibration and extraction were carried out during the GC run of the previous sample in order to reduce overall analysis time.

## Results and discussion

### Fiber properties

PAL SPME Arrow is based on a stabilizing stainless steel inner rod that runs continuously through the entire fiber, carrying the cylindrically shaped sorption phase and connecting the upper parts of the device to its solid tip, which is shown in Fig. S2 of the ESM, and specially designed to allow gentle penetration of injector and sample vial septa. This tip also retains the sorption phase, which is attached to the inner rod, and furthermore enables PAL SPME Arrow’s capability to enclose this sorption phase during transfer processes. This is an important difference to the traditional SPME fiber, which only allows for the retraction of the latter, with its outer capillary more open to external, potentially adverse influences such as contaminations from ambient air. Furthermore, an open capillary faces significant resistance during penetration processes, in contrast to a PAL SPME Arrow in its closed state. Its outer capillary rests against the solid tip, resulting in a homogeneously closed fiber since both parts possess the same diameter.

A sketch of a conventional SPME fiber and a PAL SPME Arrow is shown in Fig. 1. Pictures comparing the points and sorption phases of both instruments are included in the ESM (Figs. S1 and S2).

Classical SPME fibers can cause coring of injector septa due to their open tubular tip [2]. Based on own experiences, exchange of the septa of gas chromatographic systems, which are subject to regular SPME measurements, is required after approximately 100 injections to avoid leakages and introduction of septum material into the liner.

Using PAL SPME Arrow, the wear of injector septa diminished due to the specially designed tip. Despite the enlarged diameter compared to the classical fiber, at least 200 injections without coring, abrasion, or leakage are possible.

PAL SPME Arrow demonstrated faultless mechanical reliability over the entire course of these studies. In our experience, classical SPME fibers are more fragile, typically requiring replacement after 100 to 200 injections due to bending of the fibers (an exemplary picture is included in the ESM). These values seem to be typical and are also encountered in literature [7, 9, 10]. Active agitation of the sample vial (instead of the liquid sample via stirring) by the standard PAL agitator may decrease this value even further since the fiber material is weakened by being constantly bent into alternating directions.

The main reason for this change in mechanical reliability is the increased diameter of the fibers’ outer capillary, which is 1.5 mm in contrast to approx. 0.7 mm in the case of the classical gauge 23 SPME fiber. In addition, the tip of PAL SPME Arrow not only conserves septa during penetration but thereby also lowers the resistance, which has to be overcome.

### Extraction optimization

In general, PAL SPME Arrow and classical SPME fibers require the same optimization procedure. For the here applied direct immersion (DI) extraction, the important optimization steps are evaluation of extraction time and stirring velocity [6].

**Fig. 2:**
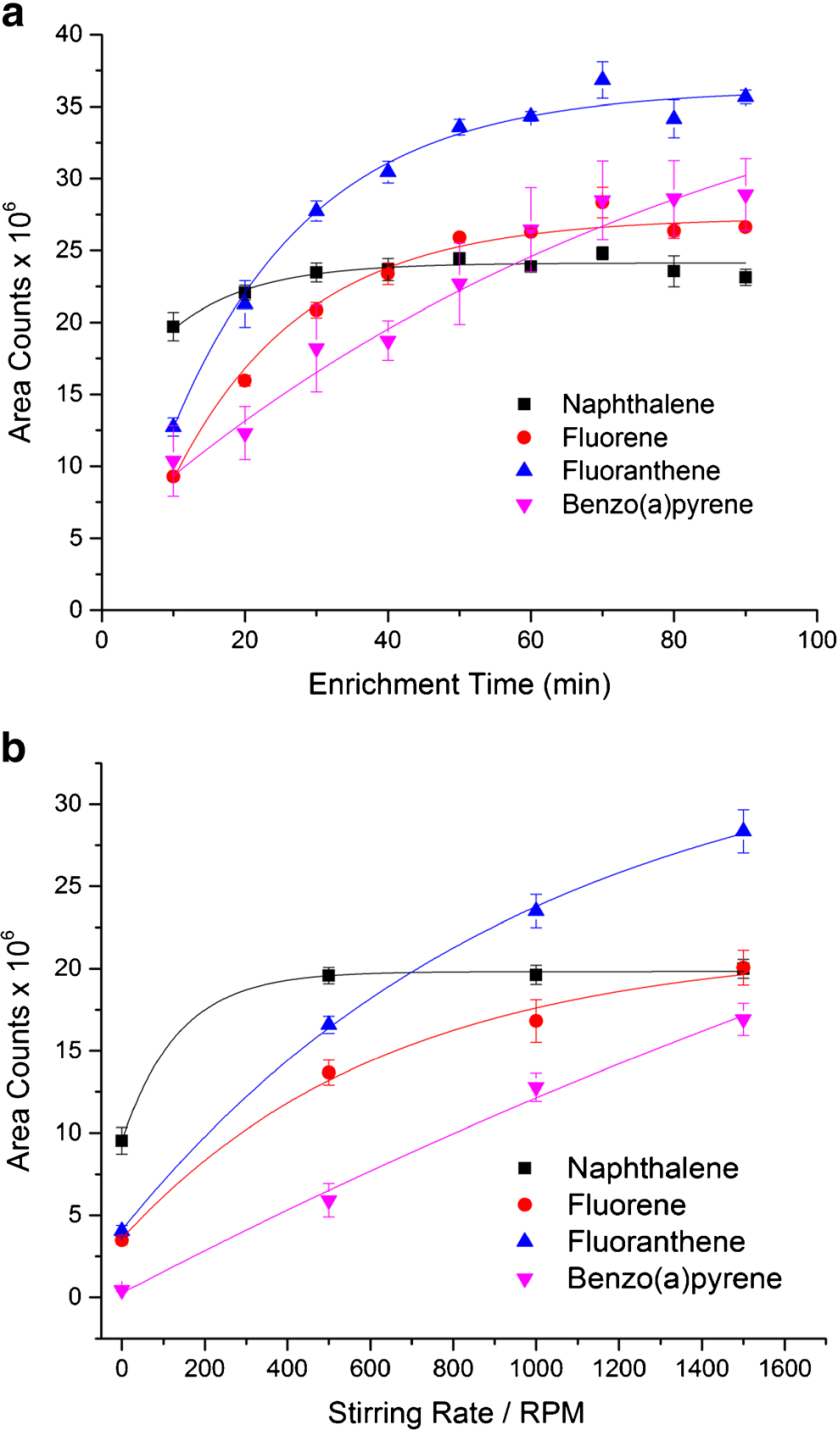
Extraction optimization measurements for PAL SPME Arrow extractions of PAH from aqueous solutions: **a** extraction time (stirring rate kept at 1500 rpm), **b** stirring rate (extraction time kept at 70 min). All samples contained 500 ng L^−1^ of PAHs and were extracted by a PAL SPME Arrow with a PDMS sorption phase (250 μm × 20 mm, 10.2 μL). *Exponential trend lines* were added via Origin Pro 2015

In Fig. 2, the influence of stirring rate and extraction time is shown exemplarily for four of the 16 EPA PAHs, with achieved results confirming expectations according to literature [2, 15, 21].

For the optimized PAL SPME Arrow method, an extraction time of 70 min was chosen. Apparently, this technique represents a reasonable compromise in this context. Classical SPME fibers typically require approx. 30 min [15] of extraction time in order to reach an equilibrium state, and alternative extraction techniques with larger sorption phases such as SBSE may require timeframes of up to 14 h [22].

In Fig. 2b, the influence of the stirring rate between 0 and 1500 rounds per minute (rpm) is shown. In accordance with the SPME extraction theory [2], an increased stirring rate leads to a higher mass transfer in the system, since the diffusion layer around the fiber coating is minimized and thus the equilibrium is attained faster. For the optimized method, the maximum possible stirring rate of 1500 rpm was used.

Since the typical behavior of decreasing extraction yields at higher temperatures caused by smaller partition coefficients of the analytes between the extraction phase and the sample matrix [2] could be observed in our preliminary measurements as well, the lowest possible temperature of 35 °C was used for all sample extractions. See Fig. S5 in the ESM for the corresponding optimization results.

### Extraction efficiency

To determine the effects of the enlarged sorption phases in the case of PAL SPME Arrow, a comparison with classical SPME fibers was performed. Prior to sample measurements, theoretically extracted analyte amounts were calculated with Eq. (1) [21]:

1 $$ {m}_f=\frac{K_{fs}{V}_f{V}_s{c}_0}{K_{fs}{V}_f+{V}_s} $$

where *m*_*f*_ is the extracted mass of analyte in the polymeric sorption phase under equilibrium conditions and *V*_*f*_ and *V*_*s*_ are the volumes of the polymer and the aqueous sample, respectively. The initial amount of each analyte present in the aqueous samples with a volume of 19 mL and an initial analyte concentration (*c*_0_) of 10 ng L^−1^ was 190 pg.

The distribution constants *K*_*fs*_ for the analytes’ phase transition from the aqueous solution into the PDMS sorption phase were calculated from literature parameters [23] and Eq. (2), yielding the results included in Table 1. The letters *E*, *S*, *A*, *B*, and *V* thereby denote the solute descriptors according to the Abraham model for excess molar refraction, dipolarity/polarizability, overall hydrogen bond acidity, overall hydrogen bond basicity, and McGowan volume, respectively [24].

**Table 1:** Calculated log *K*_*fs*_ and *m*_*f*_ values for ten exemplary PAHs included in this work, determined for a SPME fiber (100 μm × 10 mm, 0.6 μL), a PAL SPME Arrow (250 μm × 20 mm, 10.2 μL), and an SBSE bar (500 μm × 20 mm, 47 μL) for a *c*_0_ of 10 ng L^−1^, sorted by ascending log *K*_*fs*_ value, based on solute descriptors from literature [23]

Compound	log *K*_*fs*_	*m*_*f*_ (SPME fiber) (pg)	*m*_*f*_ (PAL SPME Arrow) (pg)	*m*_*f*_ (SBSE bar) (pg)	Ratio of extracted masses PAL SPME Arrow vs. SPME fiber / SBSE bar vs. PAL SPME Arrow
Naphthalene	2.8991	4.6	56.7	125.8	12.2 / 2.2
Acenaphthene	3.4196	14.6	111.2	164.7	7.6 / 1.5
Fluorene	3.6313	22.6	132.4	173.6	5.9 / 1.3
Anthracene	3.8933	37.6	153.5	180.7	4.1 / 1.2
Fluoranthene	4.2939	72.8	173.6	186.2	2.4 / 1.1
1,2-Benzanthracene	4.9443	139.7	186.1	189.1	1.3 / 1.0
Benzo(a)pyrene	4.9744	142.2	186.3	189.2	1.3 / 1.0
Benzo(b)fluoroanthene	5.0941	151.4	189.2	189.4	1.2 / 1.0
Benzo(ghi)perylene	5.6407	177.2	187.9	189.8	1.1 / 1.0
Dibenz(a,h)anthracene	5.9609	183.6	189.6	189.9	1.0 / 1.0

2 $$ \log\;{K}_{fs}=0.246+0.568E-1.305S-2.565A-3.928B+3.573V $$

According to Table 1, PAL SPME Arrows can be expected to exhibit improved extraction yields when compared to classical SPME fibers with a ratio of up to 12.2 for PAHs. In the case of the SBSE bars, the further improvement in relation to PAL SPME Arrow has a ratio of up to 2.2. Especially for molecularly larger compounds with a log *K*_*fs*_ of approx. 5 or larger, differences in extraction efficiency between PAL SPME Arrow and SBSE are negligible. Obviously, the effect of a further increase in sorption phase dimensions peaks in the range where PAL SPME Arrow is situated. The critical relation here is the phase ratio between sample and sorption phase. While these results were calculated for 20-mL vials, the SBSE technique is probably better suited for analysis of larger sample volumes, which are however less straightforward to automate.

**Fig. 3:**
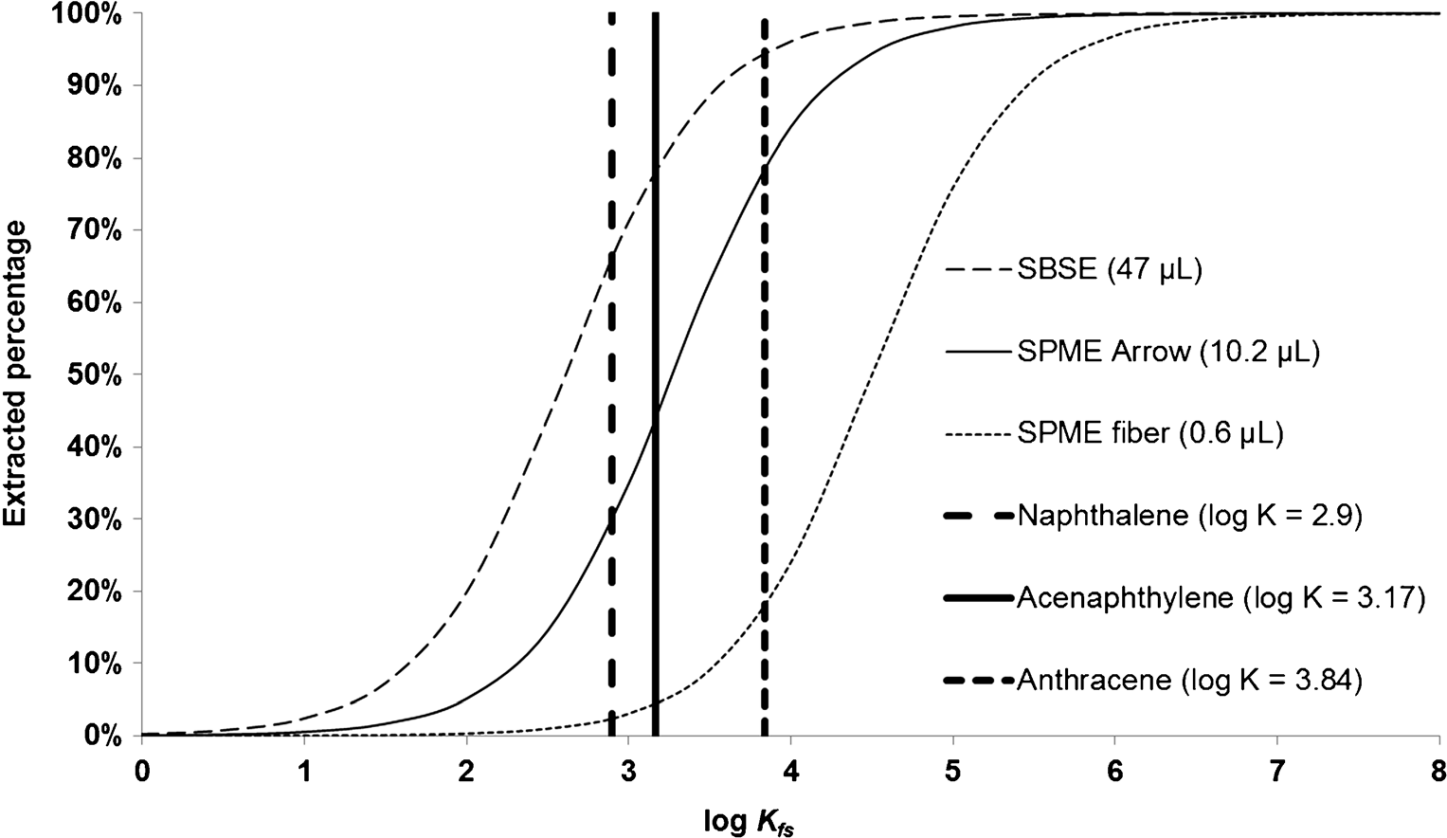
Theoretically extracted percentages for three extraction techniques and exemplary PAHs under equilibrium conditions calculated for an aqueous sample volume of 19 mL with indicated PDMS volumes and log *K*_*fs*_ values from literature [23]

Further investigation on the extraction behavior of PAL SPME Arrow was conducted by calculating the recoveries that are to be expected theoretically from PDMS-based extraction techniques with different phase volumes. We selected a commonly available variant of classical SPME fibers, a PAL SPME Arrow, and an SBSE device as representative examples. Using *K*_*fs*_ values from literature [23], we calculated the theoretically extracted percentages for the aforementioned extraction phases and three model analytes under equilibrium conditions (Fig. 3).

In order to evaluate these calculated values, the depletion SPME method [25] was used to determine the extracted percentages of analytes out of a sample with an initial concentration of 50 ng L^−1^ for a single extraction. The latter was carried out either by a classical SPME fiber (100 μm × 10 mm, 0.6 μL) or a PAL SPME Arrow (250 μm × 20 mm, 10.2 μL). This method is based on performing depletion extractions by extracting and measuring samples multiple times. The declining, logarithmical peak areas are then plotted against the number of consecutive extractions, yielding a linear regression, whose slope *b* then enables calculation of the extraction ratio *E* from log(1 − *E*) [25].

**Fig. 4:**
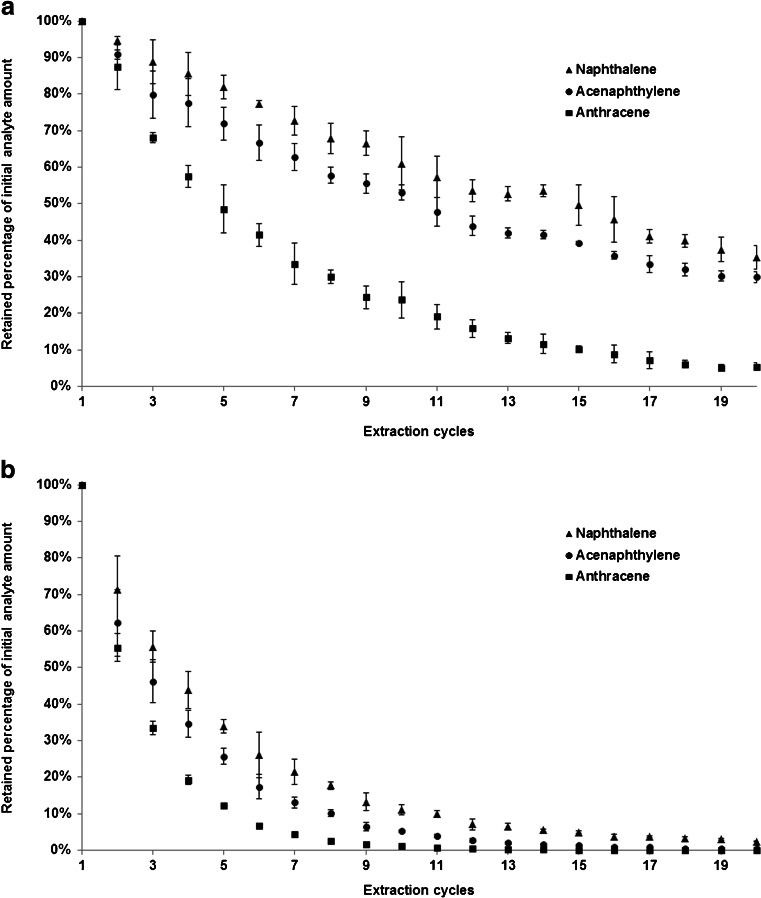
Depletion curves for three exemplary PAHs, extracted by a classical SPME fiber (100 μm × 10 mm, 0.6 μL) (**a**) and a PAL SPME Arrow (250 μm × 20 mm, 10.2 μL) (**b**)

The results of these measurements can be seen in Table 2 and are in good agreement with literature [8], as well as the previously calculated values in Table 1. This is also visible when plotting calculated against measured results with a linear trend line. An example for such plots can be found in Fig. S7 in the ESM, exhibiting a correlation coefficient of 0.9777. Depletion curves of these measurements and their corresponding linear correlations and trend lines are shown in Figs. 4 and S6. The slope of the logarithmic depletion curves and their linear correlations are also included in Table 2, demonstrating sufficiently good correlations (>0.98) for all analytes. These measurements were also carried out for the largest available PAL SPME Arrow sorption phase variant (250 μm × 30 mm, 15.3 μL), and the results are included in the ESM (Table S2).

**Table 2:** Slopes, correlation coefficients, and extracted percentages of the performed depletion experiments according to Zimmermann et al. [25] for samples containing 19 mL of water and an initial concentration of 50 ng L^−1^ PAHs for the first extraction by a classic SPME fiber (100 μm × 10 mm, 0.6 μL) and a PAL SPME Arrow (250 μm × 20 mm, 10.2 μL)

Compound	SPME fiber			PAL SPME Arrow		
	Slope	*R* ^2^	*E* (%)	Slope	*R* ^2^	*E* (%)
Naphthalene	−0.023	0.9903	5.2	−0.084	0.9915	17.5
Acenaphthylene	−0.028	0.9842	6.2	−0.134	0.9980	26.6
Acenaphthene	−0.041	0.9946	9.0	−0.144	0.9907	28.2
Fluorene	−0.050	0.9927	10.9	−0.146	0.9902	28.6
Phenanthrene	−0.060	0.9972	12.9	−0.163	0.9959	31.4
Anthracene	−0.071	0.9930	15.1	−0.225	0.9943	40.5
Pyrene	−0.097	0.9956	20.1	−0.254	0.9993	44.3
Fluoroanthene	−0.096	0.9972	19.9	−0.239	0.9996	42.3
1,2-Benzanthracene	−0.137	0.9925	27.1	−0.307	0.9946	50.7
Chrysene	−0.071	0.9235	15.1	−0.278	0.9871	43.4
Benzo(b)fluoroanthene	−0.172	0.9938	32.7	−0.317	0.9964	51.8
Benzo(k)fluoroanthene	−0.176	0.9878	33.4	−0.412	0.9937	61.3
Benzo(a)pyrene	−0.156	0.9958	30.2	−0.330	0.9854	53.2
Indeno(1,2,3 cd)pyrene	−0.159	0.9845	30.7	−0.367	0.9897	57.0
Dibenz(ah)anthracene	−0.142	0.9926	27.8	−0.465	0.9994	65.7
Benzo(ghi)perylene	−0.140	0.9967	27.6	−0.418	0.9999	61.9

The increased extraction yields of PAL SPME Arrow are also visible in Fig. 4 by comparing the depletion curves from these experiments that were either generated using a classical SPME fiber (a) or PAL SPME Arrow (b), since the latter showed a more rapid depletion of analytes in the samples.

### Comparison to literature

To enable a statistical comparison of achievable detection limits for PAL SPME Arrow with classical SPME fibers, we determined the method detection limits (MDL) according to Keith et al. [26], as well as relative standard deviations.

Using PAL SPME Arrow (250 μm × 20 mm, 10.2 μL), it was possible to calibrate in concentration ranges as low as 0.5 to 2.5 ng L^−1^ for all 16 EPA PAHs. Results are displayed in Table 3 in terms of LOD and RSD values for calibrations performed in ultrapure water and filtrated groundwater. Linear ranges and correlation coefficients for these calibrations can be found in the ESM.

**Table 3:** Calibration results obtained with PAL SPME Arrow (250 μm × 20 mm, 10.2 μL) in ultrapure water and groundwater: MDL values (calculated with a 99 % confidence interval) and relative standard deviations (RSD)

Compound	Ultrapure water		Groundwater	
	MDL (ng L^−1^)	RSD (%) (at 10 ng L^−1^)	MDL (ng L^−1^)	RSD (%) (at 10 ng L^−1^)
Naphthalene	0.3	5.7	1.2	6.9
Acenaphthylene	0.2	6.0	0.9	4.8
Acenaphthene	0.1	7.1	2.3	13.0
Fluorene	0.2	5.6	1.9	10.6
Phenanthrene	0.2	5.5	/	/
Anthracene	0.3	7.6	/	/
Pyrene	0.2	6.4	/	/
Fluoroanthene	0.2	6.2	/	/
1,2-Benzanthracene	0.1	6.2	0.7	3.8
Chrysene	0.1	11.0	0.8	4.3
Benzo(b)fluoroanthene	0.2	10.5	0.6	3.4
Benzo(k)fluoroanthene	0.2	8.6	0.6	3.2
Benzo(a)pyrene	0.3	7.2	0.5	2.4
Indeno(1,2,3 cd)pyrene	0.8	9.2	/	/
Dibenz(ah)anthracene	0.6	11.3	0.7	3.8
Benzo(ghi)perylene	0.8	11.9	0.6	3.4

In accordance to literature [27], it was impossible to determine freely dissolved PAHs via SPME fiber or PAL SPME Arrow in groundwater samples with a significant content of particulate organic matter (POM).

After removal of POM (along with sorbed compounds) via filtration, spiking of groundwater samples enabled determination of PAHs from the freely dissolved fraction with the following exceptions due to matrix interference: phenanthrene, anthracene, pyrene, fluoroanthene, and indeno(1,2,3 cd)pyrene, as indicated by the dashes in Table 3.

Table 4 displays LODs and RSDs for PAL SPME Arrow and comparable techniques. While Cheng et al. [15] extrapolated the LOD values for their classical SPME fibers from the standard deviation of their results at the lowest calibration point (10 ng L^−1^), the results presented herein were calculated from measurements at 0.5 ng L^−1^ for reagent water-based samples and at 5 ng L^−1^ for groundwater samples.

**Table 4:** MDL and RSD results obtained with PAL SPME Arrow (250 μm × 20 mm, 10.2 μL) for PAHs in water in comparison with literature data for classical SPME fibers and SBSE bars

Compound	PAL SPME Arrow		SPME (Cheng et al.) [15]		SBSE (Carrera et al.) [22]	
	MDL (ng L^−1^)	RSD (%) (at 10 ng L^−1^)	LOD (SD X 3)	RSD (conc. at S/N = 3 × 3)	LOD (conc. at S/N = 3 × 3)	RSD (%) (at 50 ng L^−1^)
Naphthalene	0.3	5.7	2.7	9.0	/	/
Acenaphthylene	0.2	6.0	1.8	6.0	0.1	/
Acenaphthene	0.1	7.1	0.9	3.0	/	/
Fluorene	0.2	5.6	3	10.0	0.1	8.3
Phenanthrene	0.2	5.5	2.1	7.0	0.1	1.1
Anthracene	0.3	7.6	2.1	7.0	0.2	2.1
Pyrene	0.2	6.4	3.6	12.0	0.2	/
Fluoroanthene	0.2	6.2	2.1	7.0	0.2	/
1,2-Benzanthracene	0.1	6.2	2.1	7.0	0.2	6
Chrysene	0.1	11.0	1.5	5.0	0.2	10.6
Benzo(b)fluoroanthene	0.2	10.5	2.7	9.0	0.1	/
Benzo(k)fluoroanthene	0.2	8.6	1.8	6.0	0.1	/
Benzo[a]pyrene	0.3	7.2	3.6	12.0	0.1	/
Indeno(1,2,3 cd)pyrene	0.8	9.2	3.6	12.0	0.3	/
Dibenz(ah)anthracene	0.6	11.3	/	/	0.3	/
Benzo(ghi)perylene	0.8	11.9	1.8	6.0	0.3	/

MDL values calculated with a 99 % confidence interval

*/* not determined

Carrera et al. [22] achieved LODs that are similar to the ones generated with PAL SPME Arrow, by extracting a 100-mL water sample for 14 h with a 500-μm × 20-mm SBSE bar. We calculated the sorption phase volume on these bars to be 47 μL, which would be approx. threefold larger as the largest available PAL SPME Arrow phase.

Determined MDLs for PAL SPME Arrow are generally more similar to those generated with the SBSE bars and approximately one order of magnitude better than those of the classical SPME fibers. In contrast to SBSE though, these results have been achieved with a fully automated method. The corresponding RSD values are thereby in the range of 5–12 % which is acceptable in such small concentration ranges and in good agreement with literature.

### Exemplary leaching experiment

For the roofing felt samples, naphthalene and acenaphthylene were the only EPA PAHs that could be measured from the freely dissolved fraction of the sample. This was expected since the material pieces inside the vials act as a second organic, hydrophobic phase. Since the sorptive properties of PAHs increase with their molecular weight, larger compounds are difficult to remove from this phase without a solvent extraction step. In addition to the two abovementioned PAHs, further compounds have been tentatively identified via their mass spectral information in the NIST library. These compounds and their estimated concentrations (converted from 300 mg to 1 g) are summarized in Table 5.

**Table 5:** Results for roofing felt extractions with ultrapure water, measured with a PAL SPME Arrow (250 μm × 20 mm, 10.2 μL): leached concentrations (estimated from calibrations for naphthalene and acenaphthylene for all other compounds) and relative standard deviations (RSD) at calculated concentrations

Compound	CAS Nr.	Concentration leached into water per gram (ng L^−1^)	RSD (%)
Naphthalene	91-20-30	15	5.2
2-Vinylnaphthalene	827-54-3	27	4.4
Biphenylene	259-79-0	14	9.4
[2-(Naphth-2-yl)vinyl]-methyl sulfone	Not available	13	4.9
1-Isoquinolinecarbonitrile	1198-30-70	24	5.2
5-Isoquinolinecarbonitrile	27655-41-0	13	4.6
Benz(a)azulene	246-02-6	16	5.1
Acenaphthylene	208-96-8	38	5.6
2,3-Naphthalenedicarbonitrile	22856-30-0	96	4.0
Diazene, 1-methoxy-2-[2-(1-naphthyl)ethenyl]-2-oxide-	Not available	14	3.3

Latter concentrations can be expected to be leached into 1 L of water, which is exposed to 1 g of roofing felt under the extraction conditions given above. Since the used calibration standards contained the 16 EPA PAHs, these results were estimated using the calibration functions of naphthalene (for naphthalene and 2-vinylnaphthalene) and acenaphthylene (for all other compounds). It should however be noted that only PAHs and structurally similar substances such as heterocycles or substituted PAHs were taken into account during these measurements.

Despite the minor concentrations recovered in this small-scale experiment, the large quantities of, e.g., bitumen-based waterproofing materials that are applied globally could still account for a significant contribution to the overall anthropogenic discharge of PAHs into the environment. Further assessment of these contributions should involve influences by temperature, acidity, and UV radiation.

## Conclusions

With PAL SPME Arrow, it was possible to measure PAHs from the freely dissolved fraction in aqueous samples down to the low nanogram-per-liter range or even below. For many compounds, this also applied if they had to be extracted from filtered groundwater. Achieved extraction yields and resulting sensitivities clearly benefit from the enlarged sorption phases of PAL SPME Arrow while all advantages of the classical SPME fiber are maintained.

As demonstrated in correlation with SBSE literature, the beneficial effect of increased sorption phase volumes declines with further increasing phase volumes, since the phase ratio between sample and sorption phase becomes less optimal unless significantly larger sample volumes in the range of liters are used. Since the handling of latter sample dimensions as well as the SBSE technique itself is more difficult to automate, PAL SPME Arrow might be a more effective solution in terms of combining maximal extraction efficiency with a fully automatable extraction device and sample size.

The only drawback of this new option in terms of the mandatory, slight widening of the injector port is considered less critical when compared to the additional thermal desorption equipment that is required for SBSE bars.

In addition, the increased mechanical robustness of PAL SPME Arrow facilitates extended, unattended measurement series typically found in routine laboratories.

Lastly, the enlarged sorption phase dimensions and the design principle of PAL SPME Arrow can be advantageous for the realization of new sorption phase materials and combinations. For instance, the enlarged surface area might enhance the effects of carbon nanomaterials, which exhibit promising potential as upcoming sorption phase materials [28, 29].

## Electronic supplementary material

ESM 1 (DOCX 1.14 mb)
