# Supplementary material for: PAL SPME Arrow—evaluation of a novel solid-phase microextraction device for freely dissolved PAHs in water
Source: Anal Bioanal Chem. 2015 Dec 16;408:943–52. doi: 10.1007/s00216-015-9187-z (PMC4709367; doi:10.1007/s00216-015-9187-z)
Supplement: Supplementary file 1 — (DOCX 1.14 mb) [file 216_2015_9187_MOESM1_ESM.docx]

Electronic Supplementary Material (ESM)

For

PAL SPME Arrow - Evaluation of a Novel Solid-Phase Microextraction Device for Freely Dissolved PAHs in Water

Andreas Kremser, Maik A. Jochmann, Torsten C. Schmidt

Instrumental Analytical Chemistry, University of Duisburg-Essen, Universitätsstraße 5, 45141 Essen, Germany

**
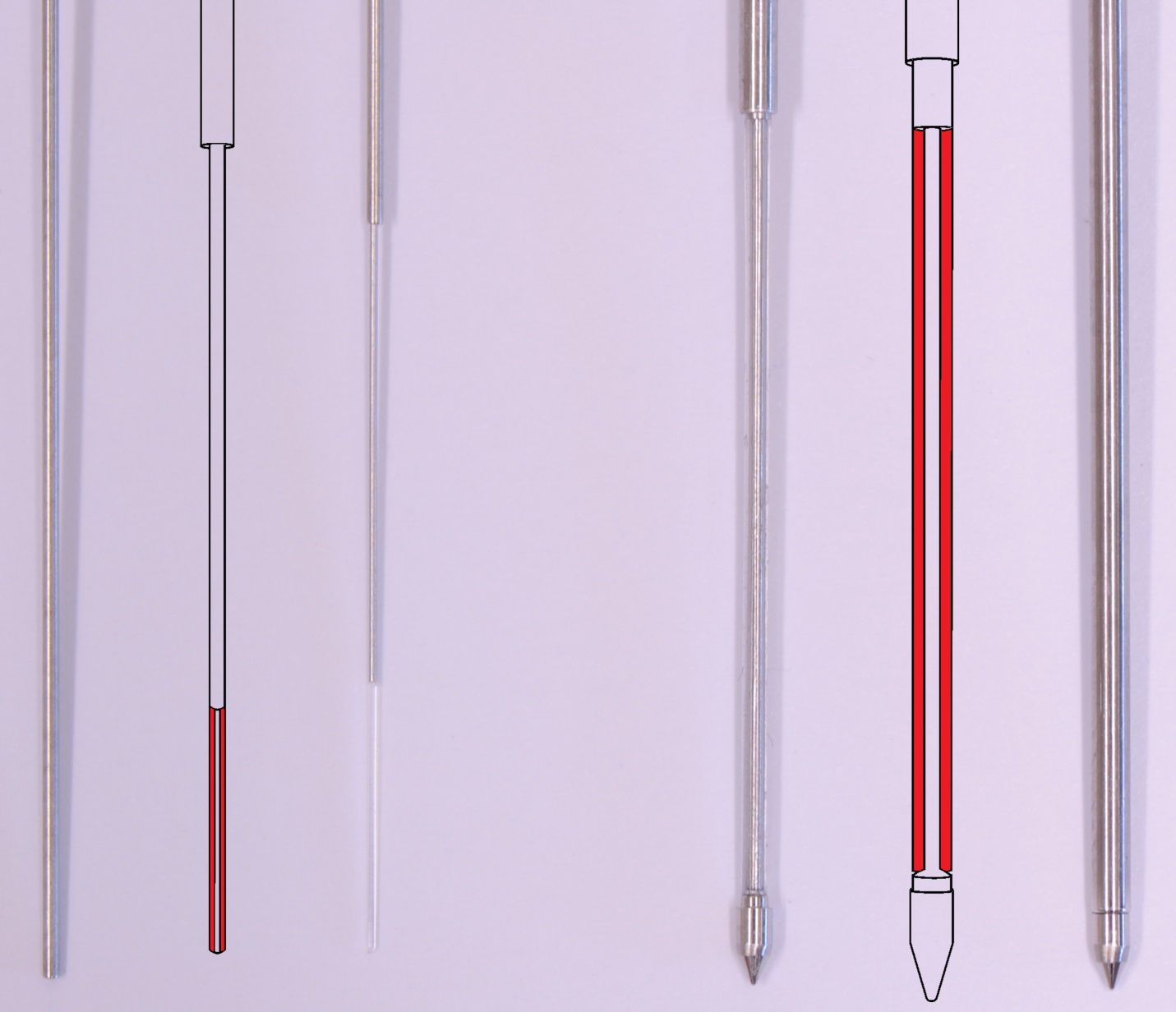
**

**Figure 1.** Pictures and sketches of a classical SPME fiber (100 µm x 10 mm PDMS phase, left) and a PAL SPME Arrow (250 µm x 30 mm PDMS phase, right), each depicted in open and closed state respectively.


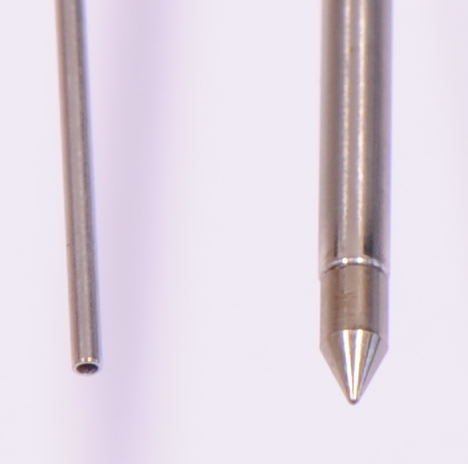


**Figure 2.** Points of a classical SPME fiber (left) and a 1.5 mm PAL SPME Arrow (right)

**
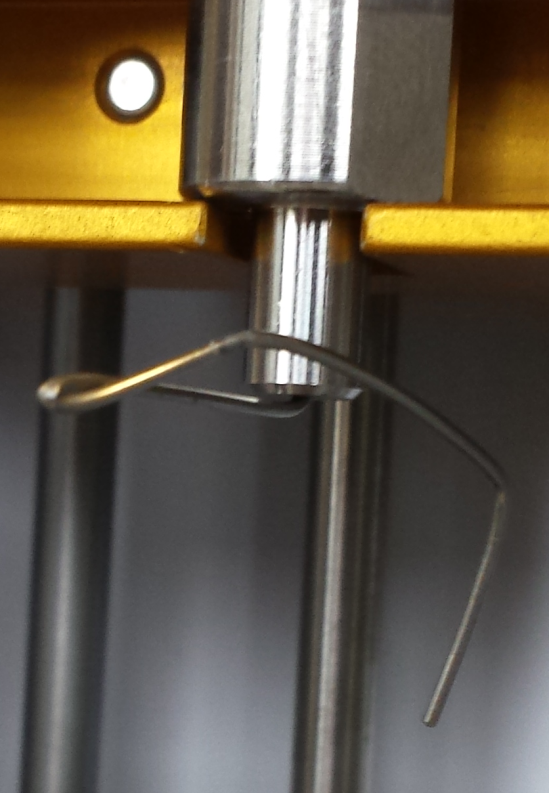
**

**Figure 3.** A bent classical SPME fiber after approximately 150 injections

**Table 1.** Constituents of the utilized EPA PAH calibration mix

| Order of elution | Analyte | CAS-Nr. |
| --- | --- | --- |
| 1 | Naphthalene D8 | 1146-65-2 |
| 2 | Naphthalene | 91-20-3 |
| 3 | Acenaphthylene | 208-96-8 |
| 4 | Acenaphthene | 83-32-9 |
| 5 | Fluorene | 86-73-7 |
| 6 | Phenanthrene | 85-01-8 |
| 7 | Anthracene | 120-12-7 |
| 8 | Pyrene | 129-00-0 |
| 9 | Fluoroanthene | 206-44-0 |
| 10 | 1,2-Benzanthracene | 56-55-3 |
| 11 | Chrysene | 218-01-9 |
| 12 | Benzo(b)fluoroanthene | 205-99-2 |
| 13 | Benzo(k)fluoroanthene | 207-08-9 |
| 14 | Benzo(a)pyrene D12 | 63466-71-7 |
| 15 | Benzo(a)pyrene | 50-32-8 |
| 16 | Indeno(1,2,3 cd)pyrene | 193-39-5 |
| 17 | Dibenz(ah)anthracene | 53-70-3 |
| 18 | Benzo(ghi)perylene | 191-24-2 |

**Chromatographic separation.** A Chromatogram of the 16 included PAHs as well as the internal standards at a concentration of 1µg L^-1^ is shown in Figure 5. The separation was accomplished on a 30 m x 0.25 mm Rxi^®^-PAH column with a 0.1 µm film thickness. Retention times varied between 8.70 min to 49.48 min for naphthalene-d_8_ and benzo(ghi)perylene (see Table 2), respectively. In accordance with literature[[1](#_ENREF_1)], the here chosen chromatographic conditions, in conjunction to the used column, enabled sufficient separation of all target compounds.


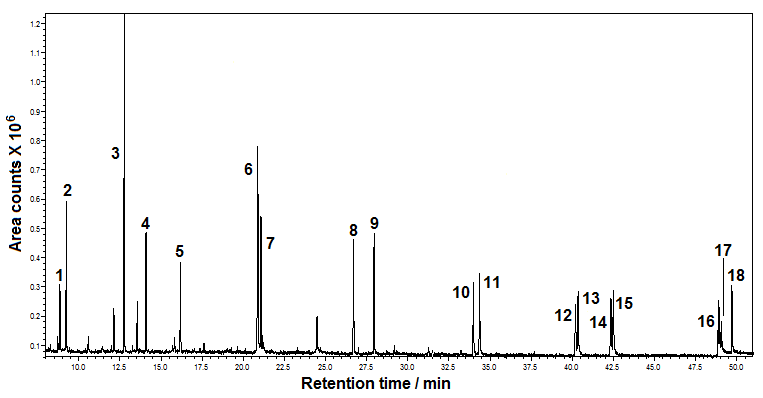


**Figure 4**. GC/MS chromatogram of standard analytes in full scan mode (m/z range: 50-350) at 1 µg L^-1^. Target compounds: (1) naphthalene-d_8_, (2) naphthalene, (3) acenaphthylene, (4) acenaphthene, (5) fluorene, (6) phenanthrene, (7) anthracene, (8) pyrene, (9) fluoroanthene, (10) benz(a)anthracene, (11) chrysene, (12) benzo(b)fluoroanthene, (13) benzo(k)fluoroanthene, (14) benzo(a)pyrene D12 (15) benzo(a)pyrene, (16) indeno(1,2,3-cd)pyrene, (17) dibenz(ah)anthracene, (18) benzo(ghi)perylene

**Mass spectrometry.** The mass spectrometer was regularly tuned with FC43 (perfluorotributylamine, CAS 311-89-7) (BGB Analytik, Boeckten, Switzerland). Ionization of analytes was performed via electron impact ionization and initial identification of PAHs was carried out in total ion current (TIC) mode in a specified m/z range of 50 to 350. Quantification of the PAHs was then conducted in selected ion monitoring (SIM) mode, using the specific m/z of ions given in Table 2. To reduce the number of simultaneously monitored m/z ratios, nine different SIM segments were used.

**Fiber variants.** PAL SPME Arrow was developed in two different variants, that either possess an outer diameter of (a) 1.5 mm, or (b) 1.15 mm. While both variants support a maximum sorption phase length of 30 mm, the thickness of such phases may be up to 250 µm in case of (a) and 100 µm in case of (b).

Compared to classical SPME fibers, both variants exhibit a significant increase in mechanical robustness and sorption phase dimensions, with a maximum polymer volume of 15.3 µL (25.5 times more volume compared to a classical 100 µm x 10 mm SPME fiber) and a maximum surface area of 84.8 mm^2^ for variant (a). The diameter of the inner rod may be varied depending on the desired extraction phase thickness and is usually 0.4 mm for the 250 µm thick phases and 0.5 mm for the 100 µm variants. The resulting outer diameters of the PDMS tubes are 0.9 mm for the 250 µm thick phase variants and 0.7 mm for the 100 µm thick phase variants.


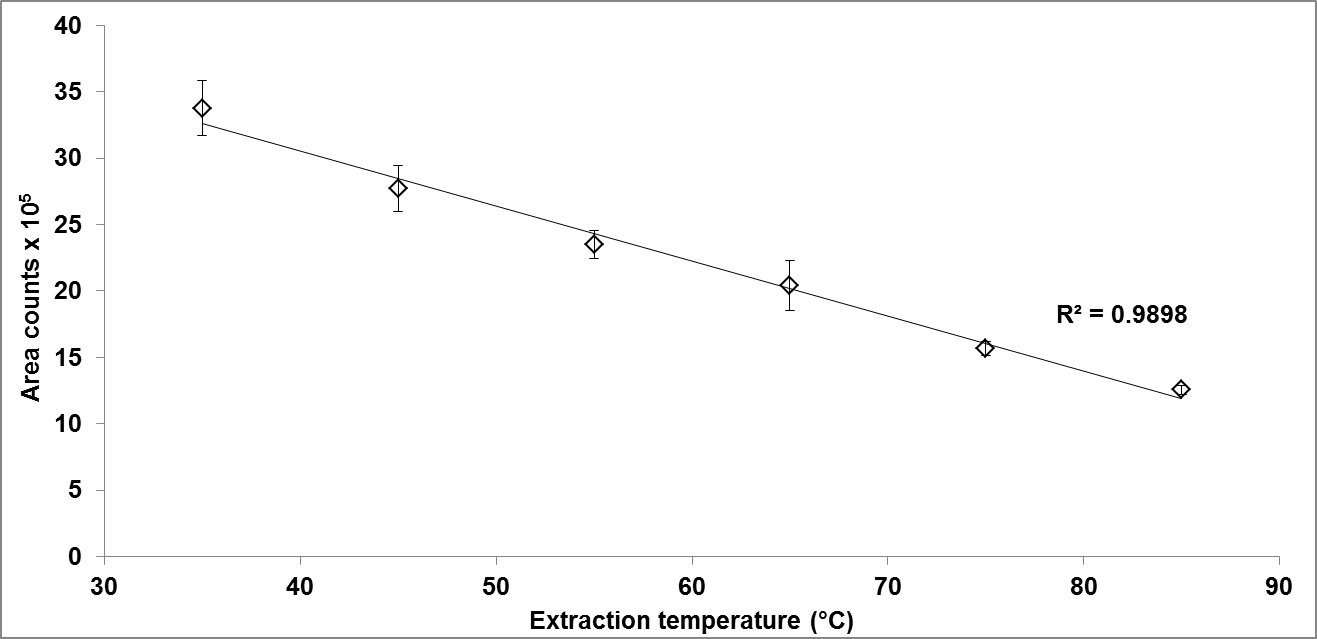


**Figure 5.** Influence of extraction temperature on anthracene peak areas determined at a concentration of 100 ng L^-1^ using a PAL SPME Arrow with a PDMS sorption phase (250 µm x 20 mm, 10.2 µL) including a linear trend line with correlation coefficient

**Table 2.** Slopes, correlation coefficients and extracted percentages determined from the performed depletion experiments according to Zimmermann et al.[[2](#_ENREF_2)] for samples containing 19 mL of water and an initial concentration of 50 ng L^-1^ PAHs for the first extraction by a classic SPME fiber (0.6 µL) and a PAL SPME Arrow (15.3 µL)

| Compound | SPME fiber | | | PAL SPME Arrow | | |
| --- | --- | --- | --- | --- | --- | --- |
|  | Slope | R^2^ | E (%) | Slope | R^2^ | E (%) |
| Naphthalene | -0.023 | 0.9903 | 5.2 | -0.184 | 0.9950 | 33.5 |
| Acenaphthylene | -0.028 | 0.9842 | 6.2 | -0.254 | 0.9997 | 44.3 |
| Acenaphthene | -0.041 | 0.9946 | 9.0 | -0.326 | 0.9981 | 52.8 |
| Fluorene | -0.050 | 0.9927 | 10.9 | -0.324 | 0.9979 | 52.5 |
| Phenanthrene | -0.060 | 0.9972 | 12.9 | -0.285 | 0.9947 | 48.1 |
| Anthracene | -0.071 | 0.9930 | 15.1 | -0.395 | 0.9908 | 59.7 |
| Pyrene | -0.097 | 0.9956 | 20.1 | -0.317 | 0.9945 | 51.8 |
| Fluoroanthene | -0.096 | 0.9972 | 19.9 | -0.290 | 0.9991 | 48.7 |
| 1,2-Benzanthracene | -0.137 | 0.9925 | 27.1 | -0.358 | 0.9901 | 56.1 |
| Chrysene | -0.071 | 0.9235 | 15.1 | -0.278 | 0.9840 | 47.3 |
| Benzo(b)fluoroanthene | -0.172 | 0.9938 | 32.7 | -0.646 | 0.9940 | 77.4 |
| Benzo(k)fluoroanthene | -0.176 | 0.9878 | 33.4 | -1.199 | 0.9916 | 93.7 |
| Benzo(a)pyrene | -0.156 | 0.9958 | 30.2 | -0.689 | 0.9998 | 79.6 |
| Indeno(1,2,3 cd)pyrene | -0.159 | 0.9845 | 30.7 | -0.739 | 0.9999 | 81.8 |
| Dibenz(ah)anthracene | -0.142 | 0.9926 | 27.8 | -1.084 | 0.9999 | 91.8 |
| Benzo(ghi)perylene | -0.140 | 0.9967 | 27.6 | -0.704 | 0.9999 | 80.2 |

**
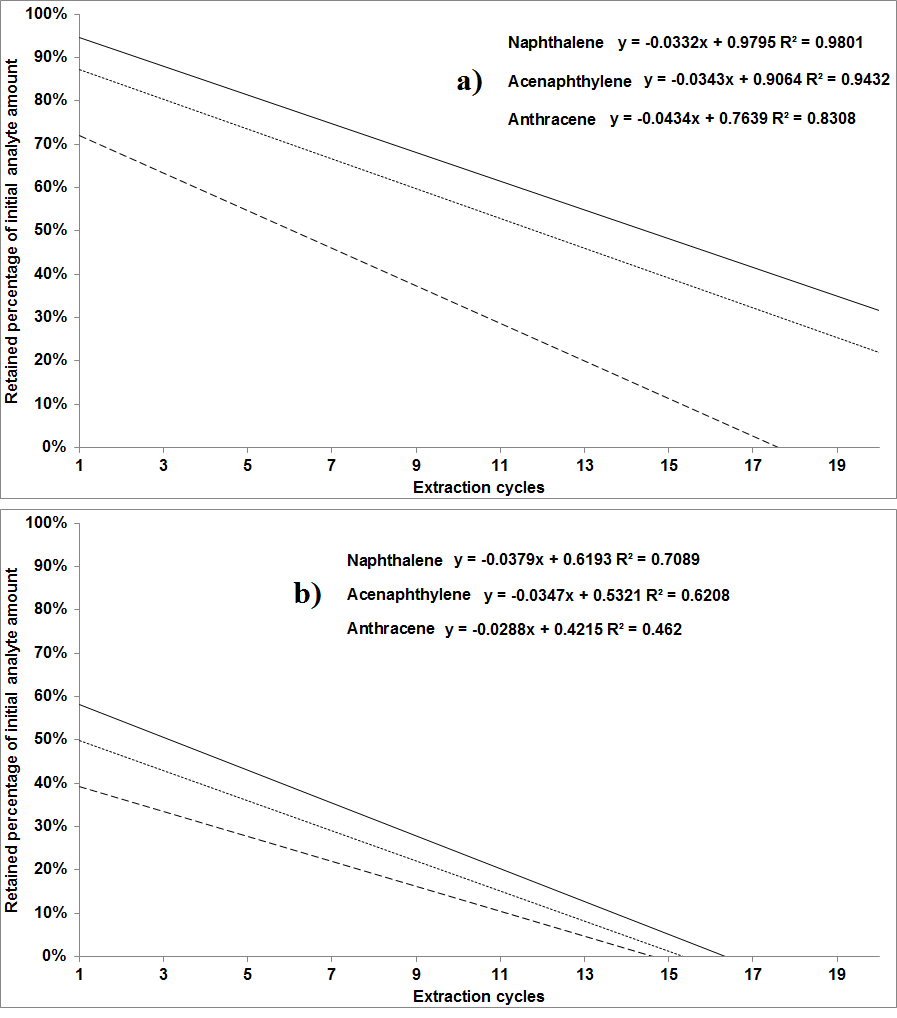
**

**Figure 6.** Linear trend lines and correlations for the depletion curves of naphthalene (continuous line), acenaphthylene (dotted line), anthracene (dashed line), extracted by a classical SPME fiber (100 µm x 10 mm, 0.6 µL) (a) and a PAL SPME Arrow (250 µm x 20 mm, 10.2 µL) (b), demonstrating more rapid exhaustion of analytes by PAL SPME Arrow

**Figure 7.** Plot of the calculated log *K_fs_* values and experimentally determined extracted percentages E (%) including a linear trend line

**Extraction capability comparison.** For an initial estimation of PAL SPME Arrows’ extraction capabilities, samples were prepared with identical analyte concentrations of 10 ng L^-1^ each and were extracted either by a classical SPME fiber (100 µm x 10 mm, 0.6 µL), or by a SPME Arrow (100 µm x 20 mm, 3.8 µL). Results are depicted in Figure 6 and show the increase in peak areas in case of the larger sorption phase volume. An approximately 2.5-fold increased extraction yield in case of SPME Arrow can be estimated from the figure. The sorption kinetics of the two fibers, in terms of required extraction times in order to reach an equilibrium state, are thereby in agreement, since the phase thickness as their main determining factor[[3](#_ENREF_3)] remains unchanged.


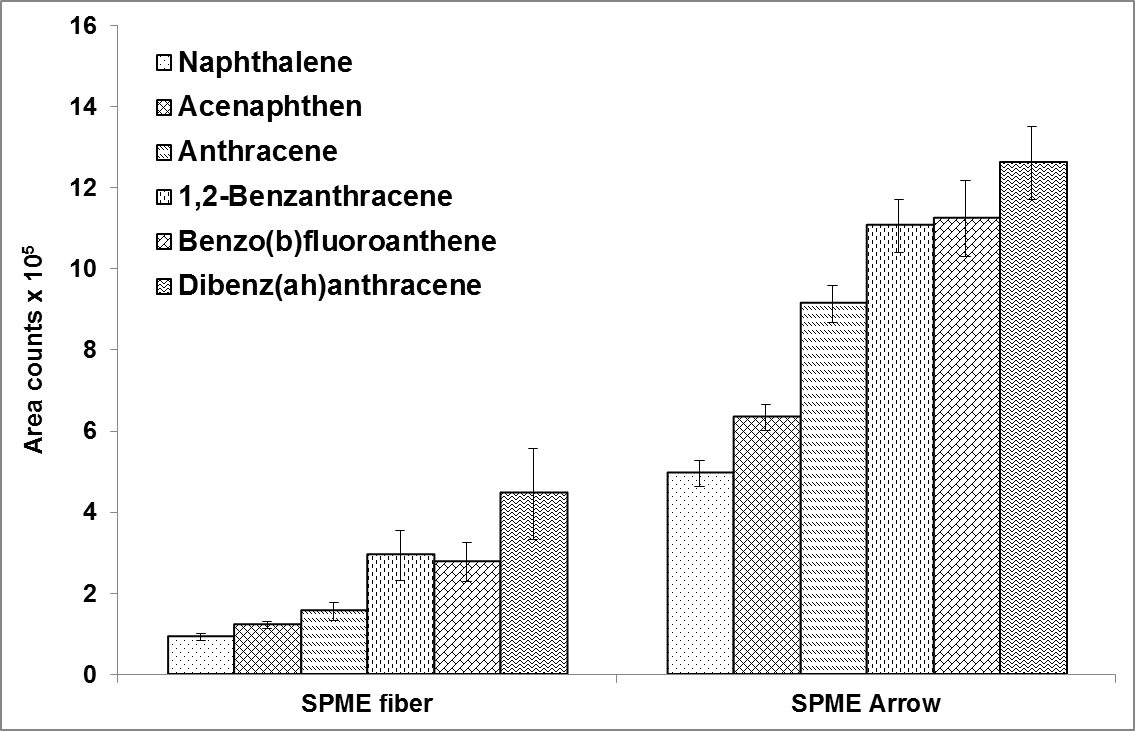


**Figure 8.** Peak area comparison for aqueous samples containing 10 ng L^-1^ of PAH each, extracted by a classical SPME fiber (100 µm x 10 mm, 0.6 µL) and a SPME Arrow (100 µm x 20 mm, 3.8 µL) via five-fold replicated immersion extraction by PDMS, shown for six exemplary PAH

**Table 3.** Calibration results obtained with a PAL SPME Arrow (250 µm x 20 mm, 10.2 µL) in ultrapure water and groundwater: Linear ranges and correlation coefficients

|  | Ultrapure water | | Groundwater | |
| --- | --- | --- | --- | --- |
| Compound | Lin. Range  (ng L^-1^) | Correlation coefficient | Lin. Range  (ng L^-1^) | Correlation coefficient |
| Naphthalene | 0.3 - 500 | 0.9878 | 1.2 - 500 | 0.9981 |
| Acenaphthylene | 0.2 - 500 | 0.9986 | 0.9 - 500 | 0.9995 |
| Acenaphthen | 0.1 - 500 | 0.9817 | 2.3 - 500 | 0.9976 |
| Fluorene | 0.2 - 500 | 0.9850 | 1.9 - 500 | 0.9861 |
| Phenanthrene | 0.2 - 500 | 0.9976 | / | / |
| Anthracene | 0.3 - 500 | 0.9966 | / | / |
| Pyrene | 0.2 - 500 | 0.9907 | / | / |
| Fluoroanthene | 0.2 - 500 | 0.9951 | / | / |
| 1,2-Benzanthracene | 0.1 - 500 | 0.9905 | 0.7 - 500 | 0.9943 |
| Chrysene | 0.1 - 500 | 0.9917 | 0.8 - 500 | 0.9909 |
| Benzo(b)fluoroanthene | 0.2 - 500 | 0.9965 | 0.6 - 500 | 0.9949 |
| Benzo(k)fluoroanthene | 0.2 - 500 | 0.9949 | 0.6 - 500 | 0.9982 |
| Benzo(a)pyrene | 0.3 - 500 | 0.9994 | 0.5 - 500 | 0.9983 |
| Indeno(1,2,3 cd)pyrene | 0.8 - 500 | 0.9939 | / | / |
| Dibenz(ah)anthracene | 0.6 - 500 | 0.9990 | 0.7 - 500 | 0.9997 |
| Benzo(ghi)perylene | 0.8 - 500 | 0.9941 | 0.6 - 500 | 0.9998 |


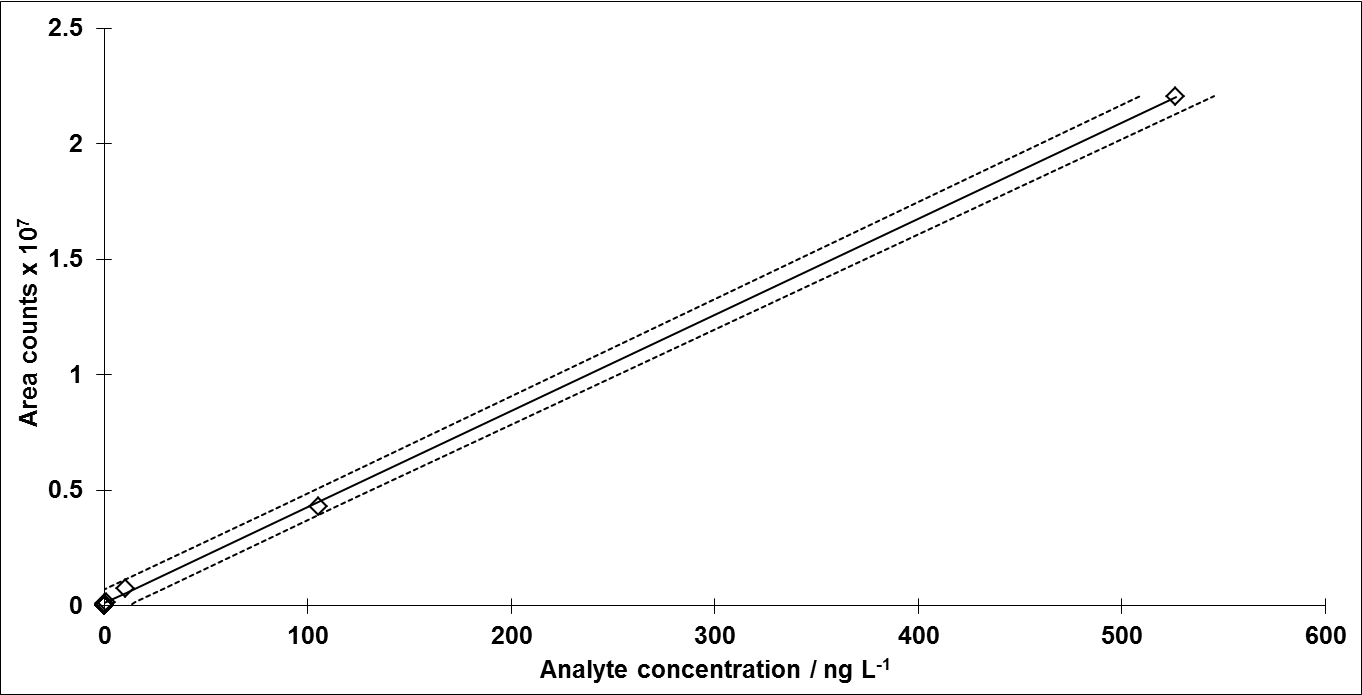


**Figure 9.** Exemplary regression line for the linear dynamic range test of naphthalene. Correlation coefficient: 0.99989, Linearity test value (F-Test according to Mandel): 1.66 with F: 98.5 (passed)

**Literature**

1. Barco-Bonilla N, Romero-González R, Plaza-Bolaños P, Fernández-Moreno JL, Garrido Frenich A, Martínez Vidal JL (2011) Comprehensive analysis of polycyclic aromatic hydrocarbons in wastewater using stir bar sorptive extraction and gas chromatography coupled to tandem mass spectrometry. Anal Chim Acta 693 (1–2):62-71

2. Zimmermann T, Ensinger WJ, Schmidt TC (2006) Depletion solid-phase microextraction for the evaluation of fiber-sample partition coefficients of pesticides. J Chromatogr A 1102 (1–2):51-59

3. Louch D, Motlagh S, Pawliszyn JB (1992) Dynamics of Organic Compound Extraction from Water Using Liquid-Coated Fused Silica Fibers. Anal Chem 64:1187-1199
